# Supplementary material for: Long-term symptom profiles after COVID-19 vs other acute respiratory infections: an analysis of data from the COVIDENCE UK study
Source: eClinicalMedicine. 2023 Oct 6;65:102251. doi: 10.1016/j.eclinm.2023.102251 (PMC10721552; doi:10.1016/j.eclinm.2023.102251)
Supplement: Appendix [file mmc1.pdf]

Long-term symptom profiles after COVID-19 versus other acute respiratory infections: an analysis of data from the COVIDENCE UK study

Giulia Vivaldi, Paul E Pfeffer, Mohammad Talaei, Tariro Jayson Basera, Seif O Shaheen, Adrian R Martineau

Supplementary appendix

Table of contents

Study recruitment and make-up 2

Table S1: COVIDENCE UK participants characteristics according to response to January, 2021, questionnaire and subsequent inclusion in analysis 2

Figure S1: Study flow diagram 3

Regression analysis 4

Table S2: Comparison between findings of ordered logistic regression models and partial proportional odds models 5

Figure S2: Symptom prevalence by infection status 6

Figure S3: Symptom prevalence by infection timing 7

Figure S4: Symptom prevalence by infection severity 8

Table S3: Raw coefficients for changes in FACIT-13 score and EQ-5D VAS 9

Table S4: Symptom associations by severity of infection, among participants with previous SARS-CoV-2 infection 10

Sensitivity analyses 11

Table S5: Symptom comparisons among participants with test-confirmed previous SARS-CoV-2 infection *vs* non-COVID-19 ARI or no infection 12

Table S6: Symptom associations by time since infection, among participants with test-confirmed previous SARS-CoV-2 infection 13

Table S7: Symptom associations by severity of infection, among participants with test-confirmed previous SARS-CoV-2 infection 14

Table S8: Symptom comparisons among participants with test-confirmed previous SARS-CoV-2 infection, non-COVID-19 ARIs, or no infection, adjusted for pre-infection general health 15

Table S9: Symptom associations by infection status, among participants with previous infection more than 12 weeks prior 16

Table S10: Symptom associations by infection status, among participants with previous infections between 28 days and 260 days prior 17

Latent class analysis 18

Table S11: Fit statistics for initial latent class model for participants with previous SARS-CoV-2 infections 18

Figure S5: BIC plot for initial latent class models 18

Figure S6: BIC plot for three-class SARS-CoV-2 models with direct effects 19

Table S12: Average latent class posterior probabilities of the final models 19

Figure S7: BIC plots for non-COVID-19 ARI and no infection models 20

Table S13: Conditional probabilities or mean severity scores for all symptoms 21

Table S14: Participant characteristics by symptom cluster for SARS-CoV-2 model 23

Table S15: Participant characteristics for the most severe symptom clusters, by infection status 25

References 26

Study recruitment and make-up

The COVIDENCE UK launch and recruitment was designed to ensure adequate representation of at-risk groups identified early on in the pandemic, such as older people, people with various comorbidities, and minoritised ethnicities. This involved multiple techniques, such as

- launching the study in newspapers such as *The Daily Express* and *The Mirror*, whose readers have an average age of 60 years or higher;
- coordination with organisations or charities, such as the Black Learning Achievement and Mental Health Charity, Asthma UK, Diabetes UK, British Lung Foundation, Primary Immunodeficiency UK, Mumsnet, Vasculitis UK, Parkinson's UK, British Liver Trust, Alzheimer's Society, British Obesity Society, KT Network, Social Action for Health, and Cancer Research UK;
- Facebook advertising targeting specific demographics;
- appearances on Bengali TV to address British Bangladeshis;
- and partnership with ReviveFM, a community radio station based in Newham that aims to bridge the gap between marginalised communities.

These approaches were taken in tandem with features in regional news programmes or websites, marketing through partner universities, printed posters in London, and use of Twitter.

Characteristics of the cohort as a whole were largely similar to the subset analysed here.[1] Participants who did not respond to the January, 2021, questionnaire share similar characteristics to those who eventually dropped out of the cohort:[1] they were younger, more likely to be male, more ethnically diverse, and more socioeconomically deprived. Among those who responded to the questionnaire, the greatest difference between those included and not included in the analysis was healthcare worker status; this is because healthcare workers were likely to report multiple respiratory infections, which excluded them from our analysis.

	**Did not respond to questionnaire**	**Responded to questionnaire**		
		All	Included	Not included
Age, years	49.6 (37.3-60.1)	62.1 (52.3–69.1)	62.8 (53.7-68.8)	60.4 (49.9-70.1)
<30	12.5%	3.4%	3.1%	3.9%
30 to <40	17.5%	5.9%	5.3%	7.2%
40 to <50	21.1%	11.6%	10.3%	14.0%
50 to <60	23.7%	22.6%	22.0%	23.7%
60 to <70	16.3%	34.3%	38.9%	25.7%
≥70	8.9%	22.2%	20.5%	25.3%
Sex				
Female	66.0%	70.5%	68.6%	73.8%
Male	34.0%	29.5%	31.4%	26.2%
Ethnicity				
White	88.8%	95.0%	95.3%	94.5%
Mixed/multiple/other ethnic groups	4.2%	2.7%	2.6%	3.1%
South Asian	5.1%	1.7%	1.5%	2.0%
Black/African/Caribbean/Black British	2.0%	0.6%	0.6%	0.5%
Quartiles of IMD decile				
Q4 (least deprived)	25.2%	32.1%	32.5%	31.5%
Q3	26.0%	25.9%	26.1%	25.6%
Q2	20.9%	20.5%	20.6%	20.4%
Q1 (most deprived)	27.9%	21.4%	20.8%	22.5%
Frontline worker				
No	72.2%	81.5%	86.7%	71.8%
Non-health	14.9%	10.4%	10.9%	9.6%
Health	12.9%	8.1%	2.4%	18.6%
Highest educational level attained				
Primary or secondary	10.9%	10.5%	11.4%	9.0%
Higher or further (A levels)	15.7%	14.5%	15.1%	13.5%
College or university	44.1%	44.3%	43.9%	45.0%
Post-graduate	29.2%	30.7%	29.6%	32.4%

Table S1: **COVIDENCE UK participants characteristics according to response to January, 2021, questionnaire and subsequent inclusion in analysis**

Figure S1: **Study flow diagram**

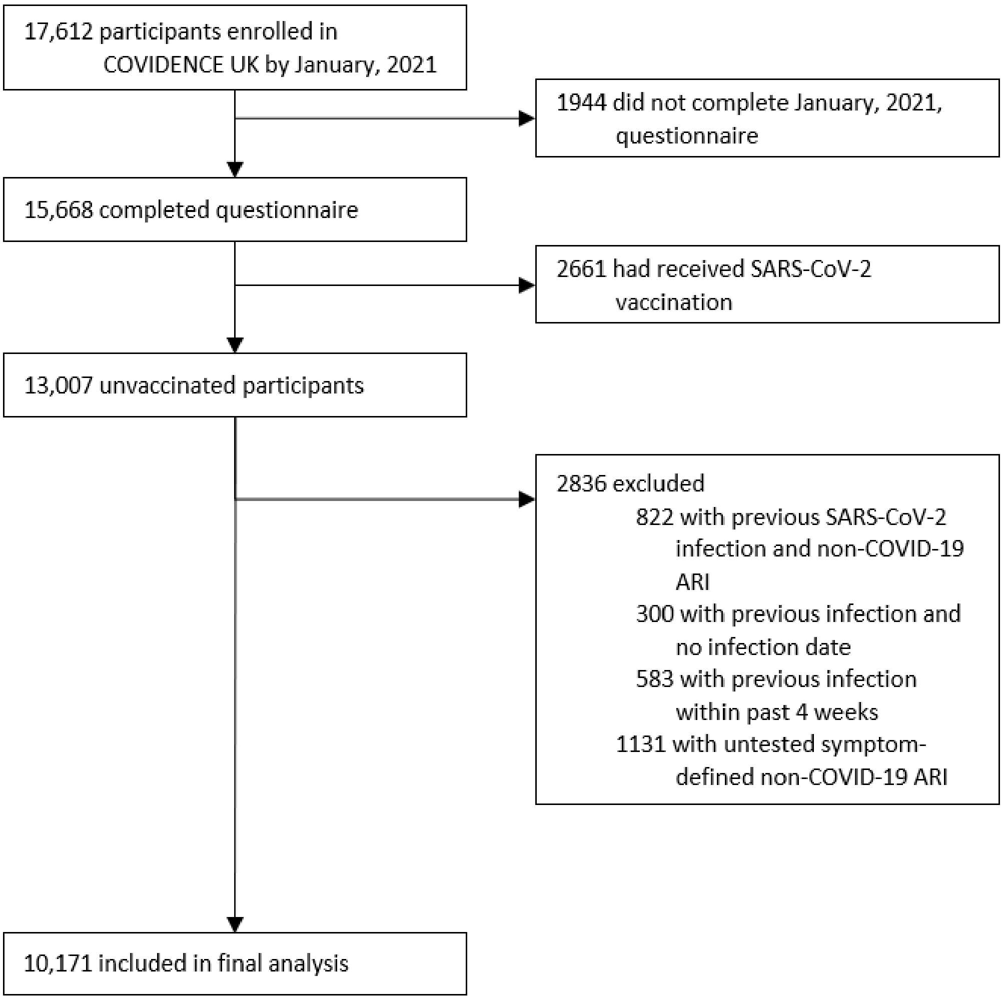

Regression analysis

Our outcome measures were a mix of binary (eg, presence or absence of symptoms), ordinal (eg, symptoms measured on a discrete scale), and continuous bounded (eg, symptom scores ranging from 0 to 100). We therefore used different regression techniques to model each of these outcomes.

	**Description**
Logistic regression	• Measures the probability of an event occurring<br>• Output can be presented as a single odds ratio
Ordinal logistic regression	• Extension of the logistic regression model to outcomes with more than two ordered categories<br>• Output can be presented as a single odds ratio, which represents the difference between *any two sequential categories*<br>• Relies on the proportional odds assumption that the effect coefficient β of a given explanatory variable is the same across all levels of the outcome (in practical terms, that the odds ratio between any two sequential categories will be the same)
Partial proportional odds model	• Can be used when the proportional odds assumption is not satisfied<br>• Sits between ordinal regression and the generalised ordered logit model<br>• Allows some—but not necessarily all—of the β coefficients to vary across different levels of the outcome variable, depending on whether they violate the proportional odds assumption<br>• A more parsimonious approach (ie, requires fewer parameters) than removing proportional odds constraints from all variables in the model
Fractional regression	• An alternative to linear regression for bounded variables, defined on the unit interval [0,1]; in such cases, linear regression may not be appropriate, and can yield problems when predictions for the response variables approach the interval boundaries<br>• Useful when boundary values are common (eg, when reporting no symptoms)<br>• Estimates are very similar to linear regression in the cases that linear regression does not present a problem

Assessment of proportional odds assumption

The proportional odds assumption for ordinal variables was assessed using the Brant test[2] (where this could not be run, a likelihood ratio test between unconstrained and constrained generalised ordered logit models was used). Models with significant deviations were refitted using a partial proportional odds model.[3] As deviations are common with larger sample sizes, model fit was assessed by comparing Lacy's adjusted ordinal explained variation measure, R^2_Oadj,[4] between the ordered logistic regression model and the partial proportional odds model. As partial proportional odds models require a greater number of parameters to be estimated, we only examined those models with a greater than 5% increase in R^2_Oadj (note: *not* percentage-point increase) to preserve model parsimony.[4]

Nine partial proportional odds models presented with more than a 5% increase in R^2_Oadj when compared with their respective ordered logistic regression models: eight for PHQ-4 and one for EQ-5D Activities. The maximum increase in R^2_Oadj was 10%. The covariate of interest (ie, infection status, infection timing, or infection severity) did not violate the proportional odds assumption in any of the models considered, and so all models produced one odds ratio for a change in level of the dependent variable. Odds ratios and 95% CIs for the covariate of interest were identical for three of the models considered. Where estimates differed, we used findings from the partial proportional odds model. The six models with different findings are listed in table S1.

	**Change in R_O^2adj**	**OR (95% CI)**	
		Ordered logistic regression	Partial proportional odds
**Main analysis**			
Non-COVID-19 ARI *vs* no infection (PHQ-4)	8%	1.34 (1.09–1.66)	1.34 (1.09–1.65)
COVID-19 severity (EQ-5D Activities)	7%	1.57 (0.65–3.79)	1.56 (0.65–3.79)
		2.22 (0.96–5.18)	2.24 (0.96–5.23)
		3.02 (1.32–6.88)	2.85 (1.25–6.50)
		13.49 (4.83–37.68)	16.25 (5.59–47.29)
**Sensitivity analysis 1**			
COVID-19 *vs* no infection (PHQ-4)	7%	1.18 (0.96–1.46)	1.19 (0.96–1.46)
COVID-19 >12 weeks prior *vs* no infection (PHQ-4)	8%	1.13 (0.89–1.44)	1.14 (0.90–1.45)
**Sensitivity analysis 2**			
COVID-19 >12 weeks prior *vs* no infection (PHQ-4)	8%	0.87 (0.53–1.44)	0.88 (0.53–1.45)
Non-COVID-19 ARI *vs* no infection (PHQ-4)	8%	1.31 (1.05–1.62)	1.30 (1.05–1.61)

OR=odds ratio. PHQ=Patient Health Questionnaire.

Table S2: **Comparison between findings of ordered logistic regression models and partial proportional odds models**

Figure S2: **Symptom prevalence by infection status**

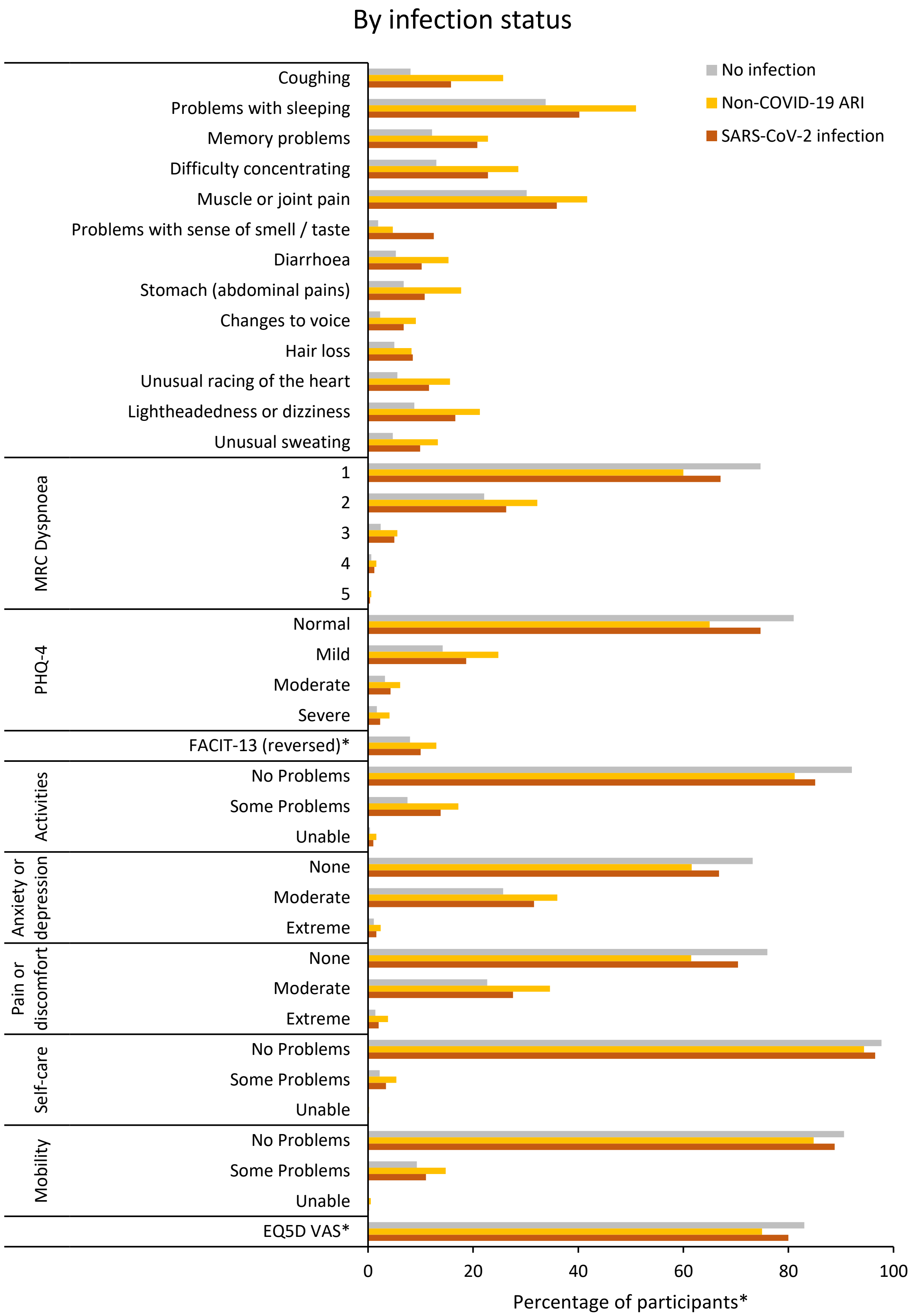

VAS=visual analogue scale. *Median scores are presented for continuous variables. FACIT-13 has been rescaled and reversed, so that 0 represents no fatigue and 100 represents maximum fatigue. For EQ-5D VAS, 0 represents worst possible health and 100 represents perfect health.

Figure S3: **Symptom prevalence by infection timing**

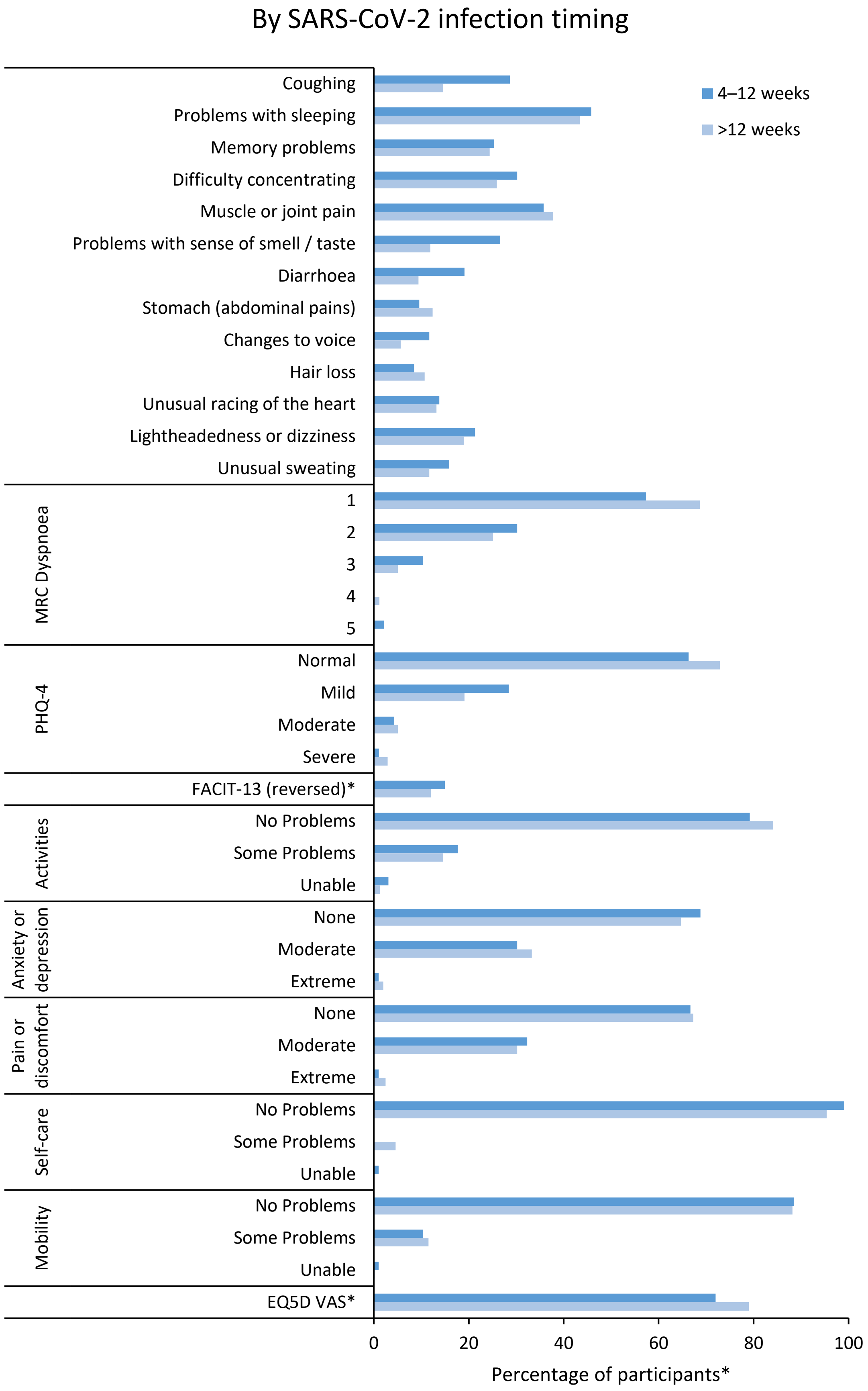

VAS=visual analogue scale. *Median scores are presented for continuous variables. FACIT-13 has been rescaled and reversed, so that 0 represents no fatigue and 100 represents maximum fatigue. For EQ-5D VAS, 0 represents worst possible health and 100 represents perfect health.

Figure S4: **Symptom prevalence by infection severity**

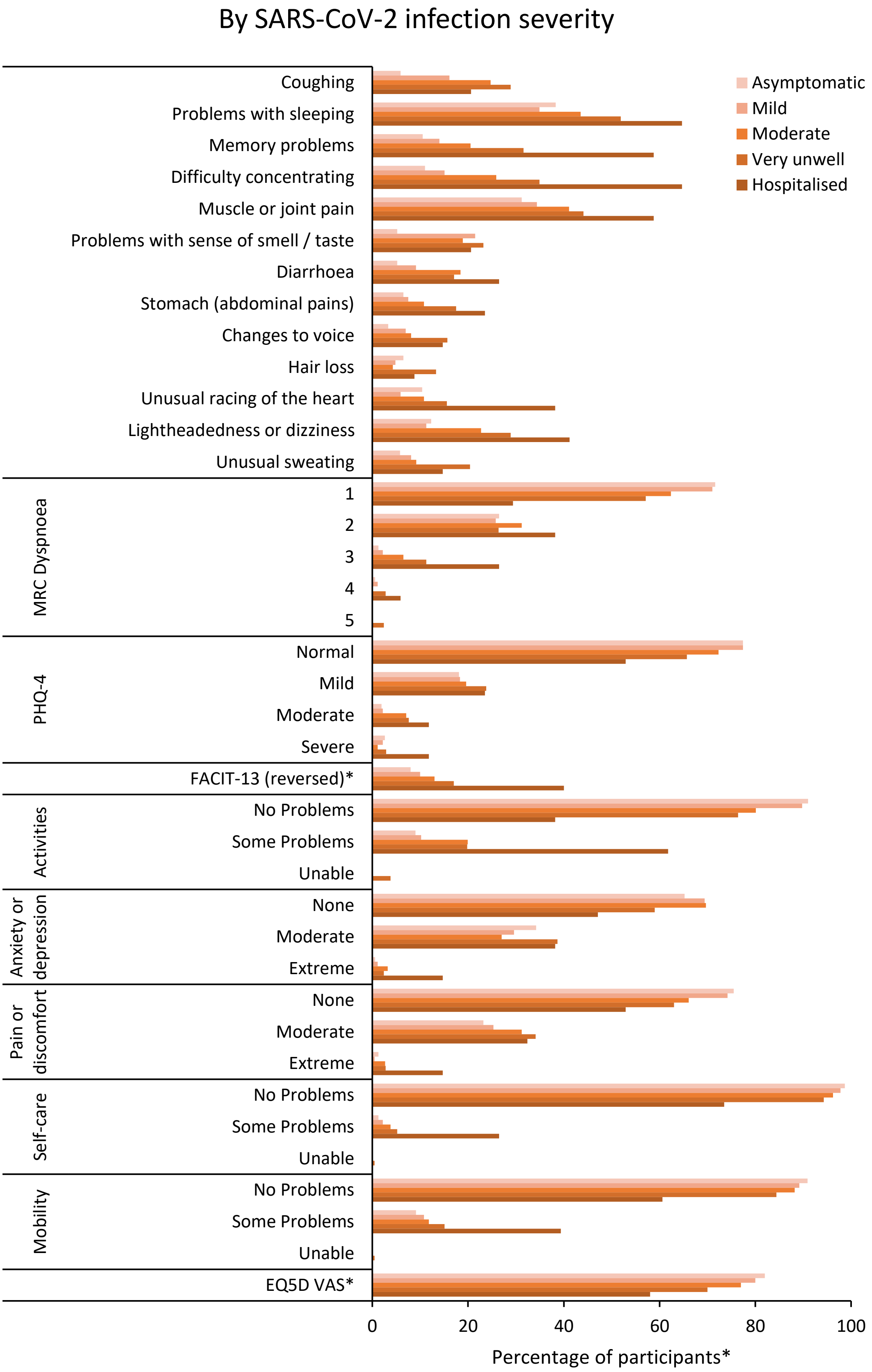

VAS=visual analogue scale. *Median scores are presented for continuous variables. FACIT-13 has been rescaled and reversed, so that 0 represents no fatigue and 100 represents maximum fatigue. For EQ-5D VAS, 0 represents worst possible health and 100 represents perfect health.

	FACIT-13 score (reversed)	EQ-5D VAS
**By infection status (table 1)**		
Previous SARS-CoV-2 infection *vs* no infection	0.31 (0.24 to 0.39)	-0.11 (-0.16 to -0.06)
SARS-CoV-2 infection >12 weeks prior *vs* no infection	0.27 (0.19 to 0.35)	-0.09 (-0.15 to -0.04)
Previous SARS-CoV-2 infection *vs* non-COVID-19 ARI	0.17 (-0.01 to 0.35)	-0.09 (-0.21 to 0.04)
Previous non-COVID-19 ARI *vs* no infection	0.33 (0.22 to 0.44)	-0.09 (-0.17 to 0.00)
**By SARS-CoV-2 infection timing (table 2)**		
SARS-CoV-2 infection >12 weeks prior *vs* 4–12 weeks prior	-0.29 (-0.49 to -0.08)	0.10 (-0.05 to 0.25)
**By SARS-CoV-2 infection severity (table S3)**		
Mildly unwell *vs* asymptomatic	0.22 (-0.08 to 0.52)	-0.18 (-0.43 to 0.07)
Moderately unwell *vs* asymptomatic	0.26 (-0.05 to 0.57)	-0.01 (-0.25 to 0.24)
Very unwell *vs* asymptomatic	0.60 (0.29 to 0.92)	-0.16 (-0.40 to 0.08)
Hospitalised *vs* asymptomatic	1.41 (0.94 to 1.87)	-0.42 (-0.76 to -0.08)

Data are estimate (95% CI). ARI=acute respiratory infection. VAS=visual analogue scale.

***Table S3:* Raw coefficients for changes in FACIT-13 score and EQ-5D VAS**

	Mildly unwell *vs* asymptomatic		Moderately unwell *vs* asymptomatic		Very unwell *vs* asymptomatic		Hospitalised *vs* asymptomatic	
	Estimate* (95% CI)	p value†	Estimate* (95% CI)	p value‡	Estimate* (95% CI)	p value§	Estimate* (95% CI)	p value‖
Coughing	3·43 (1·09–10·75)	0·035	8·38 (2·80–25·07)	<0·001	7·52 (2·53–22·36)	<0·001	6·27 (1·65–23·90)	0·007
Problems with sleep	0·76 (0·43–1·35)	0·351	1·10 (0·62–1·96)	0·736	1·04 (0·60–1·81)	0·892	2·10 (0·87–5·05)	0·099
Memory problems	1·77 (0·78–4·03)	0·170	2·66 (1·21–5·87)	0·015	4·18 (1·94–9·00)	<0·001	14·28 (5·13–39·72)	<0·001
Difficulty concentrating	2·80 (1·20–6·52)	0·017	3·66 (1·60–8·37)	0·002	5·12 (2·29–11·46)	<0·001	19·98 (6·87–58·14)	<0·001
Muscle or joint pain	0·92 (0·50–1·67)	0·776	1·01 (0·56–1·83)	0·979	1·49 (0·84–2·64)	0·174	2·97 (1·22–7·23)	0·017
Problems with sense of smell / taste	7·98 (2·93–21·72)	<0·001	6·74 (2·46–18·42)	<0·001	9·04 (3·36–24·27)	<0·001	13·50 (4·07–44·81)	<0·001
Diarrhoea	2·44 (0·75–8·01)	0·140	3·72 (1·18–11·69)	0·025	4·38 (1·43–13·48)	0·010	12·13 (3·24–45·40)	<0·001
Stomach (abdominal pains)	1·88 (0·68–5·17)	0·224	2·28 (0·85–6·10)	0·101	2·45 (0·94–6·41)	0·067	8·35 (2·62–26·58)	<0·001
Changes to voice	3·11 (0·81–11·91)	0·098	2·21 (0·56–8·71)	0·257	3·64 (0·98–13·59)	0·054	7·19 (1·47–35·15)	0·015
Hair loss	0·72 (0·26–2·01)	0·527	1·13 (0·44–2·93)	0·802	1·51 (0·61–3·72)	0·375	2·13 (0·64–7·13)	0·220
Unusual racing of the heart	0·76 (0·30–1·90)	0·553	1·01 (0·43–2·37)	0·990	1·55 (0·70–3·47)	0·282	5·92 (2·09–16·79)	<0·001
Lightheadedness or dizziness	0·97 (0·43–2·19)	0·944	2·43 (1·15–5·12)	0·020	2·19 (1·05–4·55)	0·036	4·68 (1·77–12·33)	0·002
Unusual sweating	1·17 (0·45–3·05)	0·745	1·25 (0·50–3·15)	0·633	2·53 (1·06–6·02)	0·036	1·73 (0·51–5·80)	0·377
MRC Dyspnoea¶	2·10 (1·10–4·01)	0·025	2·04 (1·07–3·90)	0·030	2·83 (1·51–5·29)	0·001	10·31 (4·38–24·27)	<0·001
PHQ-4 grade¶	0·98 (0·50–1·90)	0·947	1·17 (0·61–2·24)	0·643	1·34 (0·72–2·51)	0·360	2·59 (1·08–6·21)	0·033
FACIT-13 score (reversed)**	2·6% (–0·9 to 6·2)	0·149	3·2% (–0·5 to 6·9)	0·094	8·3% (4·2–23·8)	<0·001	23·8% (14·9–32·8)	<0·001
EQ-5D Activities¶	1·31 (0·56–3·10)	0·536	1·82 (0·81–4·11)	0·149	2·24 (1·04–4·84)	0·040	12·54 (4·59–34·27)	<0·001
EQ-5D Pain¶	0·82 (0·45–1·50)	0·524	0·84 (0·46–1·54)	0·579	0·98 (0·55–1·75)	0·955	2·18 (0·93–5·13)	0·074
EQ-5D Mobility (two groups)	0·19 (0·05–0·74)	0·017	0·14 (0·04–0·53)	0·004	0·21 (0·07–0·64)	0·006	1·00 (0·00–)	0·993
EQ-5D VAS**	2·10 (0·76–5·77)	0·151	1·41 (0·51–3·93)	0·507	1·99 (0·74–5·33)	0·171	9·07 (2·75–29·87)	<0·001

Compared with participants with an asymptomatic SARS-CoV-2 infection, those who reported mild severity of their initial infection had increased odds of problems with sense of taste or smell, whereas those who were moderately unwell also had increased odds of coughing and difficulty concentrating. Participants who were very unwell additionally had increased odds of memory problems, as well as higher levels of dyspnoea and fatigue. Participants who had been hospitalised had increased prevalence or severity of 11 symptoms or HRQoL measures. In particular, previously hospitalised participants had substantially increased odds of reporting difficulty concentrating, memory problems, and problems with sense of taste or smell. Compared with asymptomatic participants, their reported levels of fatigue were 24 percentage points higher.

Differences in EQ–5D Self Care were not estimable due to small numbers. QPHQ=Patient Health Questionnaire. MRC=Medical Research Council. VAS=visual analogue scale. *Estimates are odds ratios for binary and ordinal outcomes, and predicted percentage point changes for continuous outcomes. †After adjustment for multiple corrections, p<0·002 is the threshold for statistical significance. ‡After adjustment for multiple corrections, p<0·007 is the threshold for statistical significance. §After adjustment for multiple corrections, p<0·017 is the threshold for statistical significance. ‖After adjustment for multiple corrections, p<0·036 is the threshold for statistical significance. ¶Ordinal outcome. **Continuous outcome. Raw coefficients are shown in table S3.

Table S4: **Symptom associations by severity of infection, among participants with previous SARS-CoV-2 infection**

Sensitivity analyses

We obtained similar results in sensitivity analyses excluding participants with symptom-defined SARS-CoV-2 infection, although some analyses were affected by a lack of power (appendix tables S4–S9). When adjusting for pre-infection general health, fewer differences were observed between participants with SARS-CoV-2 infection more than 12 weeks prior and those with no infection, but odds of reporting problems with sense of taste or smell increased substantially, from 8·23 (95% CI 6·54–10·37) to 15·49 (9·00–26·65; table S8). Restricting analyses to participants with infections more than 12 weeks prior reduced differences in fatigue between the two groups, and suggested that coughing may be less likely in participants with SARS-CoV-2 infection; however, the analysis was affected by a lack of power (appendix table S9). Similarly, restricting analyses to the overlap period of infections (ie, 28 to 260 days) did not substantially change any of the point estimates, but the analysis was again affected by a lack of power (appendix table S10). Finally, we did an exploratory analysis to compare symptom prevalence and severity between participants with asymptomatic or mild SARS-CoV-2 infection and those with no infection, to examine how much our results were driven by participants with a more severe acute infection; we continued to find significant differences between the two groups for all symptoms and measures, except for self-care and mobility (data not shown).

	Previous SARS-CoV-2 infection *vs* no infection		SARS-CoV-2 infection >12 weeks prior *vs* no infection		Previous SARS-CoV-2 infection *vs* previous non-COVID-19 ARI	
	Estimate (95% CI)*	p value†	Estimate (95% CI)*	p value‡	Estimate (95% CI)*	p value§
Coughing	2.08 (1.62–2.67)	<0.001	1.50 (1.10–2.05)	0.011	1.56 (1.04–2.35)	0.033
Problems with sleep	1.23 (1.02–1.47)	0.029	1.20 (0.97–1.48)	0.086	0.86 (0.61–1.21)	0.380
Memory problems	2.12 (1.70–2.64)	<0.001	1.99 (1.55–2.56)	<0.001	1.71 (1.14–2.55)	0.009
Difficulty concentrating	1.81 (1.45–2.26)	<0.001	1.75 (1.36–2.25)	<0.001	1.37 (0.93–2.03)	0.110
Pains in muscles or joints	1.32 (1.09–1.60)	0.005	1.26 (1.01–1.57)	0.038	1.11 (0.78–1.58)	0.568
Problems with sense of smell / taste	11.98 (9.12–15.74)	<0.001	10.37 (7.62–14.11)	<0.001	18.58 (9.58–36.04)	<0.001
Diarrhoea	1.59 (1.17–2.17)	0.003	1.40 (0.97–2.02)	0.071	1.24 (0.75–2.05)	0.397
Stomach (abdominal pains)	1.58 (1.19–2.10)	0.002	1.56 (1.13–2.14)	0.007	0.84 (0.52–1.35)	0.470
Changes to voice	3.23 (2.25–4.63)	<0.001	3.06 (2.04–4.59)	<0.001	1.04 (0.55–1.97)	0.892
Hair loss	1.99 (1.47–2.70)	<0.001	2.08 (1.48–2.91)	<0.001	2.17 (1.16–4.05)	0.016
Unusual racing of the heart	2.45 (1.88–3.20)	<0.001	2.41 (1.78–3.26)	<0.001	1.46 (0.90–2.36)	0.126
Lightheadedness or dizziness	2.12 (1.67–2.69)	<0.001	2.04 (1.55–2.68)	<0.001	1.74 (1.13–2.68)	0.011
Unusual sweating	2.42 (1.82–3.22)	<0.001	2.25 (1.62–3.13)	<0.001	1.66 (0.99–2.79)	0.053
MRC Dyspnoea‖	1.56 (1.28–1.91)	<0.001	1.36 (1.08–1.70)	0.009	1.49 (1.04–2.13)	0.028
PHQ-4 grade‖	1.19 (0.96–1.46)	0.111	1.13 (0.89–1.44)	0.307	0.80 (0.55–1.15)	0.228
FACIT-13 score (reversed)¶	3.7% (2.3–5.0)	<0.001	2.6% (1.2–4.1)	<0.001	2.1% (-0.9–5.0)	0.170
EQ-5D Activities‖	2.27 (1.75–2.96)	<0.001	2.07 (1.54–2.80)	<0.001	1.39 (0.88–2.19)	0.156
EQ-5D Pain‖	1.41 (1.15–1.72)	<0.001	1.37 (1.09–1.72)	0.007	0.93 (0.64–1.35)	0.708
EQ-5D Self-care (two groups)	0.91 (0.52–1.59)	0.740	1.05 (0.59–1.88)	0.857	0.67 (0.22–2.03)	0.478
EQ-5D Mobility (two groups)	1.13 (0.83–1.54)	0.429	1.03 (0.73–1.47)	0.861	0.89 (0.51–1.54)	0.671
EQ-5D VAS¶	-2.1% (-3.5 to -0.6)	0.005	-1.6% (-3.2 to 0.1)	0.062	-1.0% (-3.9 to 1.8)	0.468

Analysis is done in 547 participants with previous SARS-CoV-2 infection (472 symptomatic), 472 participants with previous non-COVID-19 ARI, and 8388 participants with no infection. ARI=acute respiratory infection. PHQ=Patient Health Questionnaire. MRC=Medical Research Council. VAS=visual analogue scale. *Estimates are odds ratios for binary and ordinal outcomes, and predicted percentage point changes for continuous outcomes. †After adjustment for multiple testing, p<0.043 is the threshold for statistical significance. ‡After adjustment for multiple testing, p<0.036 is the threshold for statistical significance. §After adjustment for multiple testing, p<0.002 is the threshold for statistical significance. ‖Ordinal outcome. ¶Continuous outcome.

Table S5: **Symptom comparisons among participants with test-confirmed previous SARS-CoV-2 infection *vs* non-COVID-19 ARI or no infection**

	SARS-CoV-2 infection >12 weeks prior *vs* <4 weeks prior	
	Estimate* (95% CI)	p value†
Coughing	0.21 (0.12–0.38)	<0.001
Problems with sleep	1.03 (0.63–1.68)	0.921
Memory problems	0.87 (0.50–1.51)	0.622
Difficulty concentrating	0.76 (0.44–1.31)	0.326
Pains in muscles or joints	0.93 (0.57–1.53)	0.788
Problems with sense of smell / taste	0.41 (0.24–0.71)	0.001
Diarrhoea	0.50 (0.24–1.06)	0.069
Stomach (abdominal pains)	1.02 (0.49–2.14)	0.954
Changes to voice	0.66 (0.27–1.62)	0.361
Hair loss	0.89 (0.39–2.02)	0.774
Unusual racing of the heart	0.78 (0.41–1.51)	0.467
Lightheadedness or dizziness	0.98 (0.53–1.78)	0.936
Unusual sweating	0.52 (0.26–1.02)	0.055
MRC Dyspnoea‡	0.59 (0.36–0.98)	0.041
PHQ-4 grade‡	0.96 (0.57–1.62)	0.875
FACIT-13 score (reversed)§	-4.9% (-9.5 to -0.4)	0.033
EQ-5D Activities‡	0.73 (0.39–1.36)	0.656
EQ-5D Pain‡	1.13 (0.66–1.92)	0.416
EQ-5D Self-care (two groups)	1.83 (0.20–17.13)	0.370
EQ-5D Mobility (two groups)	0.71 (0.33–1.50)	0.033
EQ-5D VAS§	2.7% (-1.5 to 6.8)	0.206

PHQ=Patient Health Questionnaire. MRC=Medical Research Council. VAS=visual analogue scale. *Estimates are odds ratios for binary and ordinal outcomes, and predicted percentage point changes for continuous outcomes. †After adjustment for multiple testing, p<0.002 is the threshold for statistical significance. ‡Ordinal outcome. §Continuous outcome.

Table S6: **Symptom associations by time since infection, among participants with test-confirmed previous SARS-CoV-2 infection**

	Mildly unwell *vs* asymptomatic		Moderately unwell *vs* asymptomatic		Very unwell *vs* asymptomatic		Hospitalised *vs* asymptomatic	
	Estimate* (95% CI)	p value†	Estimate* (95% CI)	p value†	Estimate* (95% CI)	p value‡	Estimate* (95% CI)	p value§
Coughing	9.14 (1.84–45.52)	0.007	7.08 (1.43–34.96)	0.016	21.21 (4.63–97.17)	<0.001	22.31 (3.73–133.56)	<0.001
Problems with sleep	0.52 (0.23–1.15)	0.107	0.98 (0.47–2.07)	0.967	1.25 (0.63–2.50)	0.520	2.39 (0.86–6.62)	0.095
Memory problems	1.35 (0.47–3.91)	0.575	2.36 (0.91–6.10)	0.076	3.97 (1.67–9.43)	0.002	12.60 (4.04–39.26)	<0.001
Difficulty concentrating	1.25 (0.38–4.10)	0.715	2.79 (1.00–7.79)	0.051	5.93 (2.32–15.16)	<0.001	22.90 (6.70–78.31)	<0.001
Pains in muscles or joints	0.83 (0.37–1.83)	0.637	0.89 (0.42–1.92)	0.772	1.65 (0.83–3.29)	0.156	4.03 (1.49–10.90)	0.006
Problems with sense of smell / taste	4.53 (1.46–14.06)	0.009	6.18 (2.07–18.44)	0.001	7.79 (2.72–22.27)	<0.001	11.27 (3.05–41.64)	<0.001
Diarrhoea	1.24 (0.16–9.71)	0.835	7.40 (1.46–37.45)	0.015	5.62 (1.13–27.86)	0.035	26.32 (4.04–171.36)	<0.001
Stomach (abdominal pains)	1.43 (0.37–5.50)	0.598	1.85 (0.53–6.41)	0.331	2.13 (0.67–6.80)	0.203	9.09 (2.17–38.14)	0.003
Changes to voice	2.52 (0.49–12.95)	0.269	2.06 (0.41–10.43)	0.384	3.86 (0.89–16.72)	0.071	3.73 (0.59–23.51)	0.162
Hair loss	0.74 (0.19–2.88)	0.665	0.87 (0.24–3.14)	0.827	1.50 (0.51–4.45)	0.464	2.61 (0.60–11.30)	0.200
Unusual racing of the heart	0.87 (0.25–3.00)	0.822	0.75 (0.23–2.48)	0.639	1.70 (0.63–4.57)	0.293	7.01 (1.96–25.06)	0.003
Lightheadedness or dizziness	0.71 (0.20–2.58)	0.602	2.73 (1.02–7.32)	0.046	3.11 (1.23–7.85)	0.016	7.55 (2.29–24.91)	<0.001
Unusual sweating	2.46 (0.63–9.56)	0.194	0.77 (0.16–3.69)	0.739	8.99 (2.84–28.45)	<0.001	1.36 (0.24–7.74)	0.732
MRC Dyspnoea‖	1.85 (0.81–4.23)	0.147	2.09 (0.95–4.63)	0.068	2.83 (1.34–6.01)	0.007	11.29 (4.12–30.92)	<0.001
PHQ-4 grade‖	0.79 (0.32–1.98)	0.622	1.14 (0.48–2.69)	0.766	1.35 (0.62–2.95)	0.449	4.01 (1.43–11.24)	0.008
FACIT-13 score (reversed)¶	0.2% (-4.4 to 4.7)	0.947	1.5% (-3.2 to 6.2)	0.523	10.0% (4.4–28.9)	<0.001	28.9% (18.5–39.2)	<0.001
EQ-5D Activities‖	0.35 (0.07–1.81)	0.210	1.77 (0.60–5.23)	0.302	3.37 (1.32–8.62)	0.011	14.79 (4.94–44.27)	<0.001
EQ-5D Pain‖	0.96 (0.43–2.16)	0.924	0.79 (0.36–1.75)	0.563	1.03 (0.50–2.11)	0.940	3.77 (1.36–10.40)	0.010
EQ-5D Mobility (two groups)	0.89 (0.19–4.11)	0.882	1.29 (0.34–4.98)	0.710	2.30 (0.72–7.37)	0.162	15.64 (3.71–65.94)	<0.001
EQ-5D VAS¶	1.1% (-5.1 to 7.3)	0.725	-0.1% (-5.9 to 5.8)	0.983	-4.4% (-10.1 to 1.3)	0.127	-10.0% (-18.5 to -1.5)	0.021

Differences in EQ-5D Self-care were not estimable due to small numbers. PHQ=Patient Health Questionnaire. MRC=Medical Research Council. VAS=visual analogue scale. *Estimates are odds ratios for binary and ordinal outcomes, and predicted percentage point changes for continuous outcomes. †After adjustment for multiple testing, p<0.002 is the threshold for statistical significance. ‡After adjustment for multiple testing, p<0.017 is the threshold for statistical significance. §After adjustment for multiple testing, p<0.038 is the threshold for statistical significance. ‖Ordinal outcome. ¶Continuous outcome.

Table S7: **Symptom associations by severity of infection, among participants with test-confirmed previous SARS-CoV-2 infection**

	Previous SARS-CoV-2 infection *vs* no infection		SARS-CoV-2 infection >12 weeks prior *vs* no infection		Previous SARS-CoV-2 infection *vs* previous non-COVID-19 ARI		Previous non-COVID-19 ARI *vs* no infection	
	Estimate (95% CI)*	p value†	Estimate (95% CI)*	p value‡	Estimate (95% CI)*	p value§	Estimate (95% CI)*	p value‖
Coughing	2.96 (2.13–4.11)	<0.001	1.68 (0.92–3.08)	0.092	1.40 (0.91–2.16)	0.125	2.93 (2.29–3.76)	<0.001
Problems with sleep	1.46 (1.13–1.89)	0.004	1.72 (1.14–2.58)	0.010	0.94 (0.65–1.36)	0.739	1.49 (1.23–1.82)	<0.001
Memory problems	2.01 (1.46–2.77)	<0.001	1.73 (1.02–2.94)	0.043	1.44 (0.93–2.22)	0.105	1.69 (1.32–2.16)	<0.001
Difficulty concentrating	1.69 (1.23–2.33)	0.001	1.35 (0.80–2.30)	0.262	1.42 (0.94–2.16)	0.100	1.53 (1.21–1.94)	<0.001
Pains in muscles or joints	1.46 (1.11–1.92)	0.007	1.63 (1.06–2.50)	0.027	1.09 (0.74–1.61)	0.645	1.25 (1.01–1.54)	0.038
Problems with sense of smell / taste	17.63 (12.40–25.07)	<0.001	15.49 (9.00–26.65)	<0.001	19.37 (9.66–38.86)	<0.001	1.35 (0.75–2.43)	0.313
Diarrhoea	2.29 (1.53–3.41)	<0.001	1.51 (0.74–3.07)	0.256	1.42 (0.84–2.41)	0.191	2.09 (1.56–2.82)	<0.001
Stomach (abdominal pains)	1.72 (1.15–2.58)	0.008	1.79 (0.97–3.30)	0.062	0.90 (0.54–1.50)	0.681	2.15 (1.64–2.81)	<0.001
Changes to voice	3.25 (1.94–5.46)	<0.001	2.23 (0.94–5.29)	0.070	1.45 (0.76–2.76)	0.262	3.11 (2.12–4.55)	<0.001
Hair loss	1.66 (1.03–2.68)	0.038	1.46 (0.66–3.23)	0.345	1.73 (0.85–3.53)	0.131	0.97 (0.65–1.46)	0.883
Unusual racing of the heart	2.45 (1.67–3.59)	<0.001	2.38 (1.31–4.33)	0.005	1.34 (0.79–2.27)	0.282	1.79 (1.33–2.41)	<0.001
Lightheadedness or dizziness	2.40 (1.71–3.35)	<0.001	2.56 (1.53–4.28)	<0.001	1.59 (1.00–2.55)	0.052	1.54 (1.17–2.03)	0.002
Unusual sweating	2.42 (1.61–3.63)	<0.001	2.59 (1.40–4.78)	0.002	1.51 (0.85–2.67)	0.156	1.83 (1.32–2.54)	<0.001
MRC Dyspnoea¶	1.89 (1.43–2.49)	<0.001	1.51 (0.96–2.37)	0.075	1.67 (1.14–2.44)	0.008	1.36 (1.10–1.69)	0.005
PHQ-4 grade¶	1.31 (0.98–1.74)	0.072	0.87 (0.53–1.44)	0.593	0.94 (0.63–1.39)	0.745	1.30 (1.05–1.61)	0.015
FACIT-13 score (reversed)**	4.3% (2.4–6.3)	<0.001	1.8% (-0.8–4.4)	0.180	1.9% (-1.3–5.1)	0.242	3.4% (2.1–4.7)	<0.001
EQ-5D Activities¶	2.65 (1.82–3.87)	<0.001	2.17 (1.19–3.95)	0.011	1.43 (0.87–2.35)	0.163	1.79 (1.35–2.38)	<0.001
EQ-5D Pain¶	1.52 (1.14–2.03)	0.004	1.78 (1.15–2.77)	0.010	1.03 (0.69–1.54)	0.899	1.38 (1.11–1.71)	0.004
EQ-5D Self-care (two groups)	0.65 (0.25–1.70)	0.378	0.86 (0.24–3.04)	0.814	1.17 (0.38–3.57)	0.781	0.91 (0.53–1.57)	0.739
EQ-5D Mobility (two groups)	1.55 (1.00–2.39)	0.050	1.56 (0.80–3.01)	0.189	1.36 (0.76–2.43)	0.302	1.15 (0.84–1.58)	0.385
EQ-5D VAS**	-3.1% (-5.2 to -1.1)	0.003	-3.0% (-6.2 to 0.3)	0.076	-1.3% (-4.4 to 1.8)	0.421	-1.6% (-3.2 to -0.1)	0.040

Analysis is done in 260 participants with previous SARS-CoV-2 infection (185 symptomatic), 458 participants with previous non-COVID-19 ARI, and 8388 participants with no infection. ARI=acute respiratory infection. PHQ=Patient Health Questionnaire. MRC=Medical Research Council. VAS=visual analogue scale. *Estimates are odds ratios for binary and ordinal outcomes, and predicted percentage point changes for continuous outcomes. †After adjustment for multiple testing, p<0.045 is the threshold for statistical significance. ‡After adjustment for multiple testing, p<0.019 is the threshold for statistical significance. §After adjustment for multiple testing, p<0.002 is the threshold for statistical significance. ‖After adjustment for multiple testing, p<0.041 is the threshold for statistical significance. ¶Ordinal outcome. **Continuous outcome.

Table S8: **Symptom comparisons among participants with test-confirmed previous SARS-CoV-2 infection, non-COVID-19 ARIs, or no infection, adjusted for pre-infection general health**

	**SARS-CoV-2 infection >12 weeks prior *vs* non-COVID-19 ARI >12 weeks prior**	
	Estimate* (95% CI)	p value†
Coughing	0.71 (0.39–1.27)	0.250
Problems with sleep	1.02 (0.67–1.57)	0.910
Memory problems	1.63 (0.98–2.69)	0.058
Difficulty concentrating	1.34 (0.82–2.18)	0.240
Pains in muscles or joints	1.14 (0.73–1.78)	0.561
Problems with sense of smell / taste	16.67 (6.26–44.35)	<0.001
Diarrhoea	1.00 (0.52–1.90)	0.992
Stomach (abdominal pains)	1.12 (0.63–2.00)	0.702
Changes to voice	1.21 (0.51–2.87)	0.664
Hair loss	1.94 (0.90–4.20)	0.091
Unusual racing of the heart	1.27 (0.68–2.36)	0.449
Lightheadedness or dizziness	1.95 (1.15–3.32)	0.014
Unusual sweating	1.05 (0.56–1.96)	0.873
MRC Dyspnoea‡	1.36 (0.87–2.12)	0.184
PHQ-4 grade‡	0.86 (0.55–1.35)	0.506
FACIT-13 score (reversed)§	1.1% (-2.1 to 4.3)	0.494
EQ-5D Activities‡	1.55 (0.88–2.71)	0.129
EQ-5D Pain‡	1.03 (0.65–1.62)	0.909
EQ-5D Self-care (two groups)	1.42 (0.46–4.35)	0.539
EQ-5D Mobility (two groups)	0.84 (0.44–1.60)	0.598
EQ-5D VAS§	-1.2% (-4.5 to 2.0)	0.457

Analysis is done in 1101 participants with previous symptomatic SARS-CoV-2 infection and 198 participants with previous non-COVID-19 ARI. ARI=acute respiratory infection. PHQ=Patient Health Questionnaire. MRC=Medical Research Council. VAS=visual analogue scale. *Estimates are odds ratios for binary and ordinal outcomes, and predicted percentage point changes for continuous outcomes. †After adjustment for multiple testing, p<0.002 is the threshold for statistical significance. ‡Ordinal outcome. §Continuous outcome.

Table S9: **Symptom associations by infection status, among participants with previous infection more than 12 weeks prior**

	SARS-CoV-2 infection *vs* non-COVID-19 ARI	
	Estimate* (95% CI)	p value†
Coughing	1.32 (0.89–1.96)	0.174
Problems with sleep	0.93 (0.67–1.30)	0.686
Memory problems	1.65 (1.12–2.44)	0.011
Difficulty concentrating	1.49 (1.03–2.16)	0.034
Pains in muscles or joints	1.16 (0.82–1.64)	0.387
Problems with sense of smell / taste	20.83 (10.67–40.64)	<0.001
Diarrhoea	1.21 (0.75–1.97)	0.434
Stomach (abdominal pains)	0.89 (0.57–1.40)	0.622
Changes to voice	1.37 (0.77–2.43)	0.282
Hair loss	2.21 (1.20–4.04)	0.011
Unusual racing of the heart	1.32 (0.82–2.12)	0.252
Lightheadedness or dizziness	1.67 (1.10–2.53)	0.017
Unusual sweating	1.66 (1.02–2.71)	0.042
MRC Dyspnoea‡	1.45 (1.03–2.03)	0.033
PHQ-4 grade‡	0.97 (0.68–1.37)	0.842
FACIT-13 score (reversed)§	2.1% (-0.8 to 5.0)	0.161
EQ-5D Activities‡	1.43 (0.92–2.20)	0.108
EQ-5D Pain‡	0.99 (0.69–1.42)	0.972
EQ-5D Self-care (two groups)	0.82 (0.31–2.16)	0.685
EQ-5D Mobility (two groups)	1.01 (0.60–1.68)	0.983
EQ-5D VAS§	-1.5% (-4.3 to 1.2)	0.278

Analysis is done in 260 participants with previous symptomatic SARS-CoV-2 infection and 472 participants with previous non-COVID-19 ARI. ARI=acute respiratory infection. PHQ=Patient Health Questionnaire. MRC=Medical Research Council. VAS=visual analogue scale. *Estimates are odds ratios for binary and ordinal outcomes, and predicted percentage point changes for continuous outcomes. †After adjustment for multiple testing, p<0.002 is the threshold for statistical significance. ‡Ordinal outcome. §Continuous outcome.

Table S10: **Symptom associations by infection status, among participants with previous infections between 28 days and 260 days prior**

Latent class analysis
In order to choose the optimal latent class model, we first fit models including only the chosen indicators with up to ten classes. The Vuong-Lo-Mendell-Rubin test showed significant improvement in model fit with almost all additional classes, but the BIC plot showed a clear inflection point at three classes, suggesting that addition of further classes did not lead to the same improvement in model fit.

Classes	BIC	AIC(LL)	AIC3(LL)	VLMR	p value	-2LL Diff	Bootstrapped p value	Classification errors	Entropy
1	17513.6	17388.7	17412.7	··	··			0.000	1.000
2	12402.5	12163.1	12209.1	5269.6	<0.001	5269.6	<0.001	0.023	0.908
3	8554.4	8200.7	8268.7	4006.5	<0.001	4006.5	<0.001	0.023	0.948
4	8057.2	7588.9	7678.9	655.7	<0.001	655.7	<0.001	0.047	0.911
5	7501.3	6918.6	7030.6	714.3	<0.001	714.3	<0.001	0.045	0.925
6	7323.1	6625.9	6759.9	336.7	<0.001	336.7	<0.001	0.055	0.912
7	6897.7	6086.1	6242.1	583.8	<0.001	583.8	<0.001	0.055	0.920
8	6816.7	5890.6	6068.6	239.5	<0.001	239.5	<0.001	0.058	0.917
9	6528.7	5488.2	5688.2	446.4	<0.001	446.4	<0.001	0.062	0.918
10	6701.9	5546.9	5768.9	-14.8	0.962	-14.8	1.000	0.060	0.917

BIC=Bayesian Information Criterion. AIC=Akaike Information Criterion. LL=log likelihood. VLMR= Vuong-Lo-Mendell-Rubin.

Table S11: **Fit statistics for initial latent class model for participants with previous SARS-CoV-2 infections**

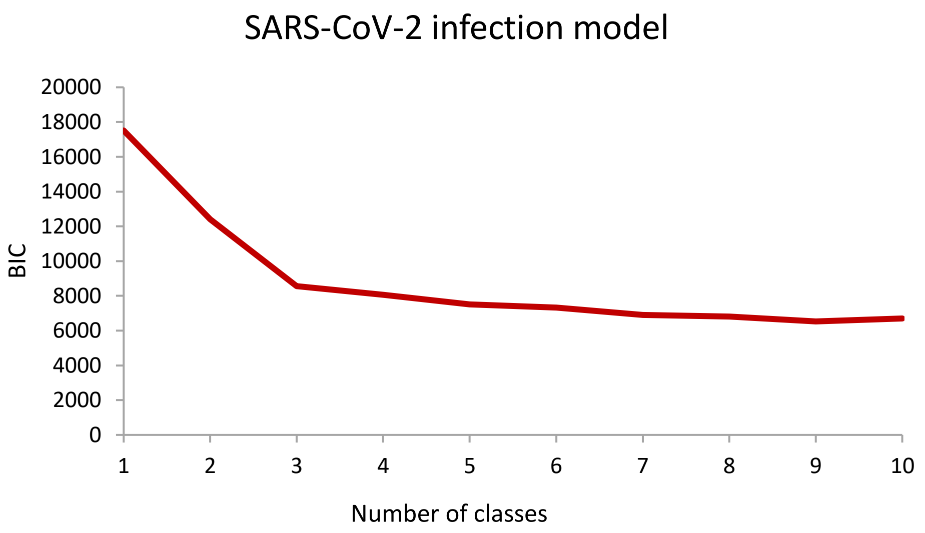

BIC=Bayesian Information Criterion.

Figure S5: **BIC plot for initial latent class models**

Additionally, examination of the profile plots showed that the class separation was less distinct in models with more than three classes, causing problems with interpretation. We therefore chose a three-class model.

The three-class model had large bivariate residuals, showing that the latent classes were not fully explaining the covariance between the included indicators. We took two approaches to improve model fit: first, we examined all pairwise correlations between variables and sequentially included direct effects for all pairs of variables with an absolute correlation of 0.5 or more, starting with the variable pair with the highest correlation; second, we sequentially included direct effects according to largest bivariate residual between variables, retaining the direct effect in the model if it improved model fit. While model fit was significantly improved by these approaches, neither approach led to a complete reduction of all bivariate residuals. To ensure parsimony, we therefore examined the BIC for signs of an inflection point and, based on this assessment, retained one direct effect between memory problems and concentration.

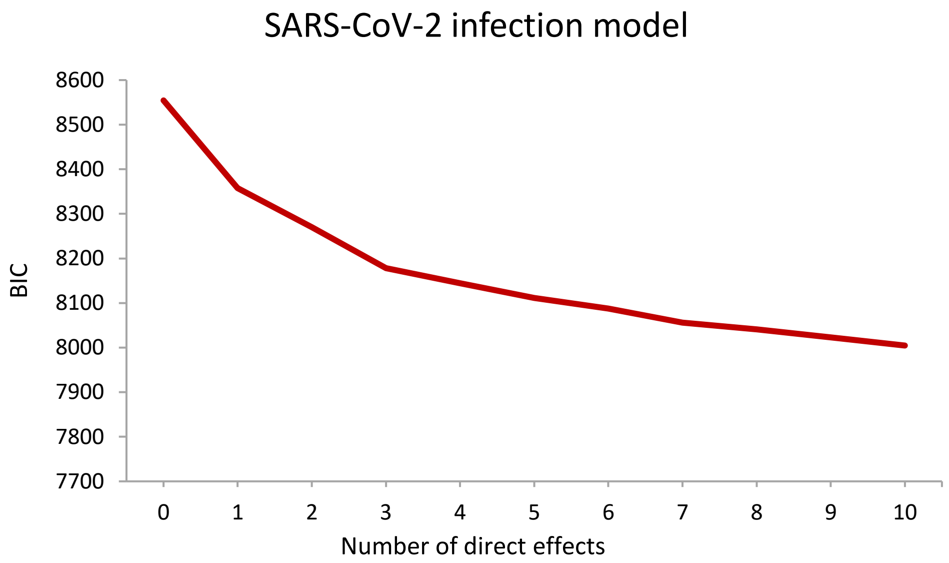

BIC=Bayesian Information Criterion.

Figure S6: **BIC plot for three-class SARS-CoV-2 models with direct effects**

Finally, to adjust for age and sex, we included each of these covariates individually in the model and examined their bivariate residuals with the indicator variables. We then added a direct effect for any pairs with a bivariate residual greater than 3.

The final model had high entropy (0.948) and showed well separated average latent class posterior probabilities.

We repeated the same procedure to establish latent class analysis models for previous non-COVID-19 ARIs and participants with no recorded infections. This led to a three-class model with two direct effects (between memory problems and concentration, and between stomach problems and diarrhoea) for non-COVID-19 ARIs, and a two-class model with one direct effect (between memory problems and concentration) for the no infection model.

	**SARS-CoV-2 model**			**Non-COVID-19 ARI model**			**No infection model**	
	Cluster1	Cluster2	Cluster3	Cluster1	Cluster2	Cluster3	Cluster1	Cluster2
Cluster1	1.000	0.000	0.000	0.999	0.000	0.000	1.000	0.000
Cluster2	0.000	0.999	0.000	0.000	1.000	0.000	0.000	1.000
Cluster3	0.000	0.000	1.000	0.001	0.000	1.000	··	··

Table S12: **Average latent class posterior probabilities of the final models**

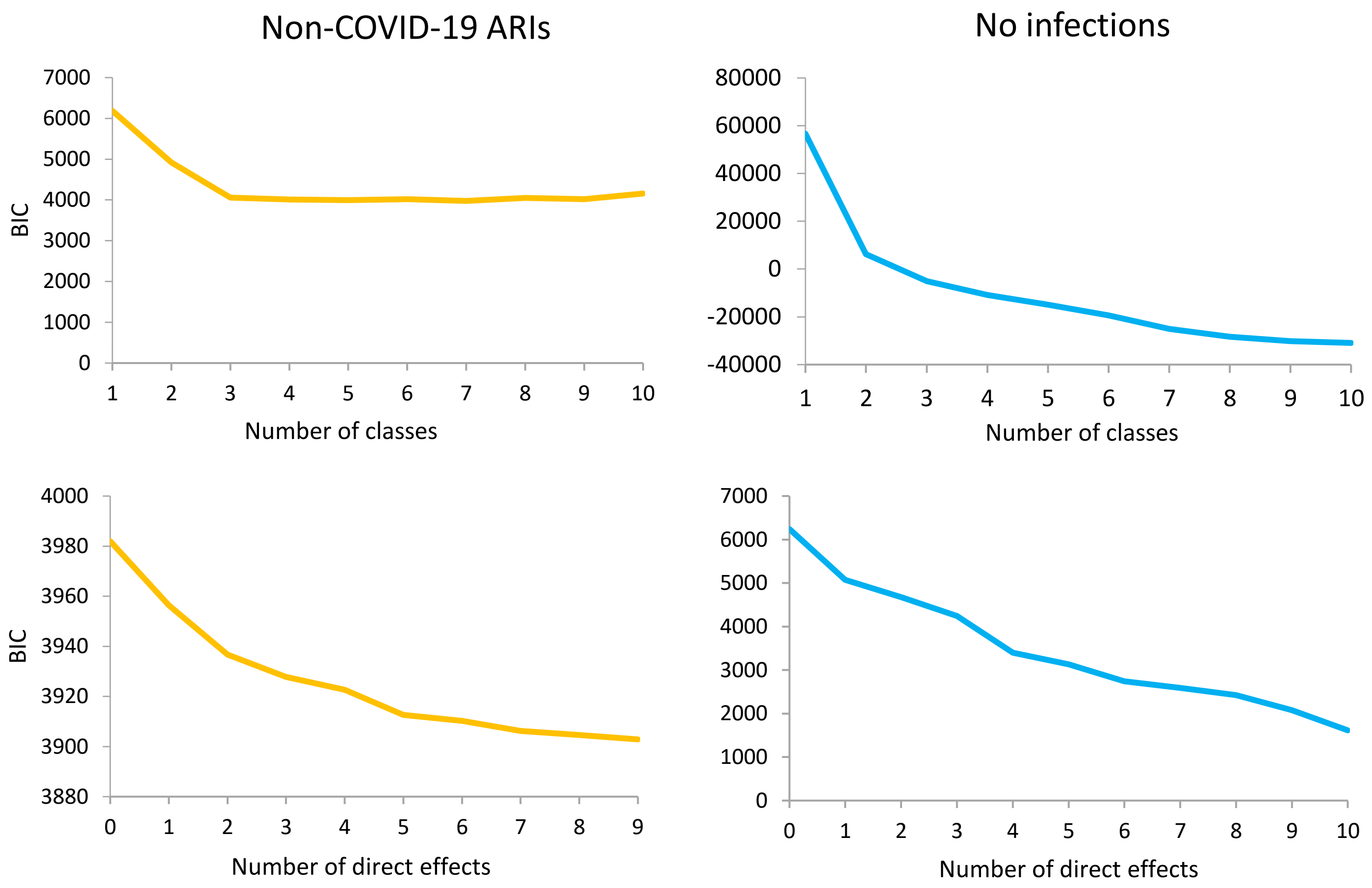

BIC=Bayesian Information Criterion.

Figure S7: **BIC plots for non-COVID-19 ARI and no infection models**

	Previous SARS-CoV-2 infection			Previous non-COVID-19 ARI			No infection	
	Mild (45%)	Moderate (32%)	Severe (22%)	Mild (40%)	Moderate (38%)	Severe (22%)	Mild (56%)	Severe (44%)
Diarrhoea	0.05	0.07	0.28	0.11	0.07	0.32	0.02	0.09
Stomach problems	0.05	0.09	0.35	0.19	0.07	0.33	0.03	0.12
Pains in muscles or joints	0.11	0.49	0.80	0.44	0.16	0.67	0.12	0.54
Sleep problems	0.24	0.44	0.77	0.52	0.31	0.78	0.21	0.51
Memory problems	0.07	0.19	0.70	0.17	0.08	0.55	0.05	0.22
Difficulty concentrating	0.06	0.20	0.77	0.23	0.07	0.65	0.05	0.24
Unusual racing of the heart	0.04	0.09	0.43	0.12	0.04	0.33	0.02	0.10
Lightheadedness or dizziness	0.06	0.15	0.56	0.10	0.06	0.48	0.04	0.16
Unusual sweating	0.04	0.08	0.38	0.11	0.04	0.25	0.02	0.08
Coughing	0.10	0.14	0.33	0.18	0.15	0.39	0.05	0.12
Changes to voice	0.03	0.04	0.20	0.07	0.04	0.20	0.01	0.04
Hair loss	0.06	0.09	0.25	0.06	0.03	0.16	0.03	0.08
Problems with smell/taste	0.09	0.11	0.41	0.02	0.02	0.04	0.01	0.03
FACIT-13†	0.07	0.13	0.44	0.15	0.09	0.46	0.06	0.17
MRC Dyspnoea‡	0.09	0.18	0.44	0.20	0.11	0.49	0.08	0.22
PHQ-4 grade‡	0.02	0.13	0.37	0.16	0.01	0.42	0.01	0.18
EQ-5D VAS†	0.19	0.27	0.44	0.27	0.21	0.44	0.18	0.29
EQ-5D Utility Index†	0.00	0.13	0.25	0.13	0.00	0.29	0.00	0.16

MRC =Medical Research Council. VAS=visual analogue scale. PHQ=Patient Health Questionnaire. *Conditional probability is shown for binary variables, and mean score for ordinal and continuous variables. †Continuous variables have been reversed to aid with interpretation, so that higher values indicate worse severity or health state. ‡Ordinal variable.

Table S13: **Conditional probabilities or mean severity scores for all symptoms**

	Mild (N=595)	Moderate (N=430)	Severe (N=286)
**Sociodemographics**			
Age, years	59.6 (50.6–65.9)	61.2 (53.3–67.4)	56.2 (48.9–63.7)
<30	23 (3.9%)	13 (3.0%)	6 (2.1%)
30 to <40	39 (6.6%)	26 (6.0%)	19 (6.6%)
40 to <50	79 (13.3%)	43 (10.0%)	59 (20.6%)
50 to <60	171 (28.7%)	116 (27.0%)	93 (32.5%)
60 to <70	205 (34.5%)	170 (39.5%)	83 (29.0%)
≥70	78 (13.1%)	62 (14.4%)	26 (9.1%)
Sex			
Female	405 (68.1%)	293 (68.1%)	210 (73.4%)
Male	190 (31.9%)	137 (31.9%)	76 (26.6%)
Ethnicity			
White	564 (94.8%)	403 (93.7%)	262 (91.6%)
Mixed/multiple/other ethnic groups	17 (2.9%)	16 (3.7%)	11 (3.8%)
South Asian	10 (1.7%)	6 (1.4%)	11 (3.8%)
Black/African/Caribbean/Black British	4 (0.7%)	5 (1.2%)	2 (0.7%)
Country			
England	544 (91.4%)	374 (87.0%)	258 (90.2%)
Northern Ireland	7 (1.2%)	7 (1.6%)	6 (2.1%)
Scotland	31 (5.2%)	30 (7.0%)	11 (3.8%)
Wales	13 (2.2%)	19 (4.4%)	11 (3.8%)
No. people per bedroom			
<1	380 (64.3%)	288 (67.1%)	140 (49.6%)
1 to <2	199 (33.7%)	130 (30.3%)	132 (46.8%)
2 to <3	11 (1.9%)	11 (2.6%)	8 (2.8%)
3 to <4	1 (0.2%)	0 (0.0%)	2 (0.7%)
Quartiles of IMD decile			
Q4 (least deprived)	209 (35.2%)	132 (30.7%)	79 (27.6%)
Q3	129 (21.7%)	105 (24.4%)	55 (19.2%)
Q2	127 (21.4%)	88 (20.5%)	56 (19.6%)
Q1 (most deprived)	129 (21.7%)	105 (24.4%)	96 (33.6%)
Frontline worker			
No	517 (86.9%)	349 (81.2%)	201 (70.3%)
Non-health	59 (9.9%)	66 (15.3%)	60 (21.0%)
Health	19 (3.2%)	15 (3.5%)	25 (8.7%)
Highest educational level attained			
Primary or secondary	50 (8.4%)	61 (14.2%)	41 (14.3%)
Higher or further (A levels)	82 (13.8%)	61 (14.2%)	41 (14.3%)
College or university	274 (46.1%)	188 (43.7%)	127 (44.4%)
Post-graduate	189 (31.8%)	120 (27.9%)	77 (26.9%)

Clinical characteristics			
BMI, kg/m^2	24.6 (22.3-27.6)	26.0 (23.5-29.8)	27.4 (24.0-31.3)
<25	320 (53.9%)	179 (41.6%)	91 (31.8%)
25 to <30	197 (33.2%)	148 (34.4%)	98 (34.3%)
≥30	77 (13.0%)	103 (24.0%)	97 (33.9%)
Asthma	89 (15.0%)	94 (21.9%)	80 (28.0%)
Atopy	152 (25.5%)	115 (26.7%)	84 (29.4%)
Autoimmune disease	35 (5.9%)	39 (9.1%)	36 (12.6%)
Cancer			
Past (cured or in remission)	51 (8.6%)	40 (9.3%)	15 (5.2%)
Active treatment	3 (0.5%)	6 (1.4%)	1 (0.3%)
COPD	11 (1.8%)	12 (2.8%)	14 (4.9%)
Diabetes	5 (0.8%)	14 (3.3%)	20 (7.0%)
Heart disease	7 (1.2%)	15 (3.5%)	11 (3.8%)
Hypertension	92 (15.5%)	98 (22.8%)	62 (21.7%)
Immunodeficiency	5 (0.8%)	2 (0.5%)	5 (1.7%)
Kidney disease	10 (1.7%)	5 (1.2%)	9 (3.1%)
Major neurological conditions	16 (2.7%)	13 (3.0%)	11 (3.8%)
Number of symptoms reported*	1 (0–2)	3 (1–4)	8 (6–10)
**Infections**			
Weeks since infection			
4–12 weeks	80 (13.4%)	42 (9.8%)	43 (15.0%)
>12 weeks	515 (86.6%)	388 (90.2%)	243 (85.0%)
Infection severity†			
Asymptomatic	46/318 (14.5%)	37/237 (15.6%)	6/197 (3.0%)
Mildly unwell	85/318 (26.7%)	44/237 (18.6%)	31/197 (15.7%)
Moderately unwell	82/318 (25.8%)	56/237 (23.6%)	40/197 (20.3%)
Very unwell	99/318 (31.1%)	94/237 (39.7%)	92/197 (46.7%)
Hospitalised	6/318 (1.9%)	6/237 (2.5%)	28/197 (14.2%)
Ever reported long COVID‡	78 (13.1%)	94/237 (21.9%)	158 (55.2%)
Reported long COVID in current questionnaire	35 (5.9%)	49 (11.4%)	137 (47.9%)

Data are n (%) or median (IQR). BMI=body-mass index. IMD=Index of Multiple Deprivation. *Does not include health-related quality of life measurements. †For symptomatic and non-hospitalised participants, severity was self-reported with the following statements: “Mildly unwell – I could do most of my usual activities”, “Moderately unwell – I couldn’t do usual activities but didn’t need to go to bed in the daytime”, and “Very unwell – I had to go to bed in the daytime”. ‡Answered ‘Yes’ to the question “Would YOU say that you currently have 'long COVID', i.e. ongoing symptoms more than four weeks after the onset of proven or suspected SARS-CoV-2 infection” before or on date of survey.

Table S14: **Participant characteristics by symptom cluster for SARS-CoV-2 model**

	**Previous SARS-CoV-2 infection (N=286)**	**Previous non-COVID-19 ARI (N=101)**	**No infection (N=3665)**
**Sociodemographics**			
Age, years	56.2 (48.9–63.7)	55.2 (42.0–63.5)	63.6 (54.7–69.5)
<30	6 (2.1%)	12 (11.9%)	119 (3.2%)
30 to <40	19 (6.6%)	11 (10.9%)	195 (5.3%)
40 to <50	59 (20.6%)	18 (17.8%)	334 (9.1%)
50 to <60	93 (32.5%)	25 (24.8%)	724 (19.8%)
60 to <70	83 (29.0%)	25 (24.8%)	1451 (39.6%)
≥70	26 (9.1%)	10 (9.9%)	842 (23.0%)
Sex			
Female	210 (73.4%)	81 (80.2%)	2629 (71.7%)
Male	76 (26.6%)	20 (19.8%)	1036 (28.3%)
Ethnicity			
White	262 (91.6%)	94 (93.1%)	3506 (95.7%)
Mixed/multiple/other ethnic groups	11 (3.8%)	4 (4.0%)	87 (2.4%)
South Asian	11 (3.8%)	2 (2.0%)	49 (1.3%)
Black/African/Caribbean/Black British	2 (0.7%)	1 (1.0%)	23 (0.6%)
Country			
England	258 (90.2%)	90 (89.1%)	3216 (87.8%)
Northern Ireland	6 (2.1%)	4 (4.0%)	63 (1.7%)
Scotland	11 (3.8%)	5 (5.0%)	232 (6.3%)
Wales	11 (3.8%)	2 (2.0%)	153 (4.2%)
No. people per bedroom			
<1	140 (49.6%)	47 (47.0%)	2481 (68.1%)
1 to <2	132 (46.8%)	49 (49.0%)	1090 (29.9%)
2 to <3	8 (2.8%)	3 (3.0%)	68 (1.9%)
3 to <4	2 (0.7%)	1 (1.0%)	2 (0.1%)
Quartiles of IMD decile			
Q4 (least deprived)	79 (27.6%)	32 (31.7%)	1088 (29.7%)
Q3	55 (19.2%)	24 (23.8%)	969 (26.4%)
Q2	56 (19.6%)	23 (22.8%)	770 (21.0%)
Q1 (most deprived)	96 (33.6%)	22 (21.8%)	837 (22.8%)
Frontline worker			
No	201 (70.3%)	80 (79.2%)	3,190 (87.2%)
Non-health	60 (21.0%)	17 (16.8%)	384 (10.5%)
Health	25 (8.7%)	4 (4.0%)	84 (2.3%)
Highest educational level attained			
Primary or secondary	41 (14.3%)	18 (17.8%)	477 (13.0%)
Higher or further (A levels)	41 (14.3%)	13 (12.9%)	593 (16.2%)
College or university	127 (44.4%)	42 (41.6%)	1593 (43.5%)
Post-graduate	77 (26.9%)	28 (27.7%)	996 (27.2%)

**Clinical characteristics**			
BMI, kg/m^2	27.4 (24.0–31.3)	27.1 (23.4–29.9)	25.7 (23.0–29.7)
<25	91 (31.8%)	38 (37.6%)	1611 (44.1%)
25 to <30	98 (34.3%)	38 (37.6%)	1172 (32.1%)
≥30	97 (33.9%)	25 (24.8%)	872 (23.9%)
Asthma	80 (28.0%)	27 (26.7%)	629 (17.2%)
Atopy	84 (29.4%)	33 (32.7%)	944 (25.8%)
Autoimmune disease	36 (12.6%)	15 (14.9%)	425 (11.6%)
Cancer			
Past (cured or in remission)	15 (5.2%)	6 (5.9%)	332 (9.1%)
Active treatment	1 (0.3%)	1 (1.0%)	39 (1.1%)
COPD	15 (5.2%)	3 (3.0%)	101 (2.8%)
Diabetes	20 (7.0%)	9 (8.9%)	224 (6.1%)
Heart disease	11 (3.8%)	10 (9.9%)	154 (4.2%)
Hypertension	62 (21.7%)	15 (14.9%)	935 (25.5%)
Immunodeficiency	5 (1.7%)	1 (1.0%)	29 (0.8%)
Kidney disease	9 (3.1%)	6 (5.9%)	87 (2.4%)
Major neurological conditions	11 (3.8%)	2 (2.0%)	124 (3.4%)
Number of symptoms reported*	8 (6–10)	7 (5–9)	2 (1–4)
**Infections**			
Weeks since infection			
4–12 weeks	43 (15.0%)	59 (58.4%)	··
>12 weeks	243 (85.0%)	42 (41.6%)	··
Infection severity†			
Asymptomatic	6 (3.0%)	··	··
Mildly unwell	31 (15.7%)	42 (41.6%)	··
Moderately unwell	40 (20.3%)	31 (30.7%)	··
Very unwell	92 (46.7%)	22 (21.8%)	··
Hospitalised	28 (14.2%)	6 (5.9%)	··
Ever reported long COVID‡	158 (55.2%)	17 (16.8%)	64 (1.7%)
Reported long COVID in current questionnaire§	137 (47.9%)	12 (11.9%)	41 (1.1%)

Data are n (%) or median (IQR). ARI=acute respiratory infection. BMI=body-mass index. IMD=Index of Multiple Deprivation. * Does not include health-related quality of life measurements. †For symptomatic and non-hospitalised participants, severity was self-reported with the following statements: "Mildly unwell – I could do most of my usual activities", "Moderately unwell – I couldn't do usual activities but didn't need to go to bed in the daytime", and "Very unwell – I had to go to bed in the daytime". ‡Answered 'Yes' to the question "Would YOU say that you currently have 'long COVID', i.e. ongoing symptoms more than four weeks after the onset of proven or suspected SARS-CoV-2 infection" before or on date of survey. §Previous non-COVID-19 ARI: 5 (2.6%) in the mild cluster and 3 (1.7%) in the moderate cluster reported long COVID. No infection: 14 (0.3%) in the mild cluster reported long COVID.

Table S15: **Participant characteristics for the most severe symptom clusters, by infection status**

References

1. Holt H, Relton C, Talaei M, et al. Cohort Profile: Longitudinal population-based study of COVID-19 in UK adults (COVIDENCE UK). *Int J Epidemiol* 2022.
2. Brant R. Assessing Proportionality in the Proportional Odds Model for Ordinal Logistic Regression. *Biometrics* 1990; **46**(4): 1171-8.
3. Williams R. Generalized ordered logit/partial proportional odds models for ordinal dependent variables. *Stata Journal* 2006; **6**(1): 58-82.
4. Lacy MG. An Explained Variation Measure for Ordinal Response Models With Comparisons to Other Ordinal R^2 Measures. *Sociological Methods & Research* 2006; **34**(4): 469-520.
